# The Foundations of interprofessional curriculum. Finding the right epistemology and learning theory for the task

**DOI:** 10.15694/mep.2017.000127

**Published:** 2017-07-12

**Authors:** C. Scott Smith, India King, Amber Fisher, William Weppner, Winslow Gerrish

**Affiliations:** 1 University of Washington; 2 Boise Center of Excellence in Primary Care Education; 3 Family Medicine Residency of Idaho

**Keywords:** Ambulatory, Complex Adaptive Systems, Constructivism/Constructivist, Reductionism/Reductionist

## Abstract

This article was migrated. The article was marked as recommended.

**Introduction**: Health professional education programs are currently focusing on interprofessional training. This can highlight differences between our professions in our learning theories and training assumptions. A standardized approach to picking a deliberate perspective from which to design specific interprofessional curricula may be useful.

**Discourses**: This paper presents one such approach. It is based on a 3 X 3 matrix developed by interprofessional faculty over seven years of team-based clinical training. To use this matrix, a deliberate epistemology (e.g., reductionist, constructivist, or complexity) and a learning theory (competency-based education, clinical reasoning, and situated learning) are chosen based on the goals of training, the context, and the developmental stage of the learners.

**Application**: Each element in the matrix then provides a focused set of considerations for designing and assessing interprofessional curriculum. In addition, this matrix provides a framework for incorporating other epistemologies and learning theories.

**Conclusions**: As professionals, we have been enculturated to see health education from a single perspective. A wider, structured approach to adopting learning assumptions and theories may better match the interprofessional training tasks we are being asked to design.

## Introduction

You have been asked to represent your training program with a group that is developing a new interprofessional curriculum for primary care clinic which will involve trainees from medicine, nursing, pharmacy, and psychology. You wonder what principles can guide you. How do other health care professionals learn about health and disease? Which learning theory is most appropriate? How will this be different than developing a single-profession curriculum? How can you thoughtfully incorporate leaners at different development levels?

Interprofessional education occurs when two or more professions learn with, about, and from each other ( CAIPE 1997). To appreciate this assignment, it is useful to understand epistemologies and learning theories as envisioned by each discipline. An epistemology is a set of assumptions about how the world is, how knowledge of the world can exist, and what procedures can distinguish between justified beliefs and opinions. Epistemologies form the foundation for learning theories, which hypothesize how knowledge is acquired, retained and used, and therefore how it should be taught. Learning theories are important because they guide instruction and assessment.

The authors base their recommendations on seven years of experience designing and improving interprofessional curriculum as part of a U.S. Department of Veterans Affairs (VA) effort to develop team-based training in a patient-centered medical home (Gilman et al., 2014). Each of us has found that it is difficult to step outside our own discourse, the epistemology and learning theories that we were enculturated into during our own professional training. However, interprofessional training forces this meta-perspective by revealing the tension between discourses (Smith et al., 2015). Teams must learn to find a shared vocabulary, develop self-awareness, and embrace differences. This is the approach that will make your response to the above scenario succeed, and is the basis of our recommendations.

In this paper, we review three important epistemologies; reductionism, constructivism, and complexity (complex adaptive systems) and explore their properties relative to three learning theories; competency-based education, clinical reasoning, and situated learning. While there are other epistemologies and learning theories, these are currently very important in health professional education. These three epistemologies are most often construed as competitive and mutually exclusive. In this paper, we propose a pragmatic approach, that these epistemologies are synergistic and should be deliberately selected as the right ‘tool’ for a given context and purpose.

## Discourses

### Epistemologies

Reductionism, constructivism, and complexity are complimentary epistemologies that will be described in detail below. While each profession has default assumptions, it is important to select the appropriate epistemology based on educational goals, the learner’s developmental level, and context

### Reducionism

Reductionism assumes that the world is: 1) real and independent of the observer; 2) orderly and uniform; and 3) operates by means of fundamental laws that are discoverable. In this epistemology, knowledge is best justified through controlled experiments. Properly identified fundamental laws are assumed to be the basis for explicit knowledge that can be used to make causative inferences and predictions ( Carnap 1963, Greenland 1998). This is a *prescriptive* epistemology, it tells you what to do. Reductionism supports a ‘vessel’ theory of learning, where the learner is ‘filled’ with facts by the teacher or text.

Reductionism is the most common epistemology in the training clinic. It supports evidence-based practice, guidelines, and other important tools. Regarding educational tasks, reductionism is best applied in situations where the clinical elements are stable and not controversial. Reductionist simplification is ideal for learners who are unfamiliar with clinic and who are often not ready to manage its full complexity. Also, reductionism is critical to the process of expanding knowledge capacity through ‘instrumentalization’, the compilation of knowledge beyond individual brains in policies, web sites, books, etc. ( Davis et al 1993, Hidalgo 2015).

However, problems with reductionism exist. Many domains of practice are not stable enough to assume unchanging facts, and knowledge may be very context dependent. To be most suitable, reductionist facts must be delivered as being useful, but potentially contingent and dynamic. For example, the ‘facts’ that were taught about estrogen replacement therapy changed radically between the early and late 1990s as research demonstrated new evidence ( Herrington 2003). There was an unwarranted concreteness conveyed in the early ‘facts’-they were assumed to be established, universal and enduring. As the evidence changed, the nuanced debates at the level of experts led to confusion at the level of practitioners and trainees. Reductionism can also imply a separation of ‘body’ and ‘mind’, which can lead to biased inferences, especially in early learners ( Gendle 2016). A good example of the proper application of reductionism is teaching learners to use equipment to obtain vital signs. For beginners, this task is unfamiliar, does not change rapidly, and is not controversial. It can be made interprofessional through a discussion of roles and responsibilities, e.g., who should obtain vital signs in what circumstances.

### Constructivism

Constructivism assumes that the world is: 1) real and linked to the observer through fallible senses, 2) context-dependent, and 3) intersubjectively determined through discourse. In this epistemology, knowledge is justified as the creation of shared meaning through dialogue. Shared meaning is tested by the interaction between concepts and real world experience. This is an *adaptive* epistemology; it leads to better and better approximations of the world. Constructivism supports a ‘searchlight’ theory of learning, where the learner seeks the most practical models for explaining the world, such as a ‘Starling curve’ model of heart failure.

Regarding educational tasks, constructivism is best applied to situations where the elements are stable but the conversation about the proper approach to these elements is dynamic or controversial. Constructivism is an increasingly important perspective for interprofessional training as it focuses on relationships, providing a link between shared meaning and normative action that allows critique of discourse. For instance, in one study of a surgical team the differences between a team member’s self-image and the description from ‘other’ professions in how they attributed motivation and responsibility created conflict ( Lingard et al 2002). Only after examining these constructs from a ‘meta-perspective’, comparing and contrasting competing discourses, can we begin to be aware of our tacit assumptions and examine our implicit biases and stereotypes.

Differences in discourse are very common in interprofessional education and important to discuss. For simplicities sake, we collapse post-structuralist analyses (Fox, 2016) into constructivism as the epistemology of choice when social forces are important (power differentials, conflict, hidden curriculum). Instrumentalized knowledge (see reductionism above), while useful for expanding knowledge beyond personal experience, can lead to informational power differentials. Those that hold the knowledge are in control, which can support hierarchical relationships and learner disparities ( Mavelli 2013).

Constructivist methods, such as facilitated discussion, provide a mechanism for understanding relationships, and are ideal for iteratively building trust in a team. They can also be used for matching and refining curriculum to the expected learner trajectory. Constructivist methods should be foundational in any longitudinal experience, such as establishing norms for respectful communication.

There are also limitations with constructivism. First, these methods are tutor-dependent and quite different than more reductionist transfer models of teaching (lecture, presentation, rounds). Faculty may not have been exposed to underlying theories and be unfamiliar with role modeling, so they may be uncomfortable in the role of ‘facilitator’ rather than ‘teacher’. It is difficult to measure an effect of constructivist learning by predominantly reductionist standards. Unfamiliar evaluative techniques, such as social network analysis, may be required. Finally, constructivist methods require authentic participation. Rigid enculturation in specific professions with different perspectives and values about knowledge, relationships, and curriculum often pose barriers to authentic participation. An example of the application of constructivist methods might be an interprofessional group discussion about the highly context-dependent and dynamic nature of treatment goals for a chronic disease.

### Complexity

Complexity assumes that the world is: 1) dynamic, 2) emergent, and 3) co-determined in conjunction with its components. In this epistemology, knowledge is evanescent and contingent, surprise is expected, and prediction is short-term at best. This is a *proscriptive* epistemology (tells you what to avoid) as opposed to a prescriptive epistemology (tells you what to do). Complexity is well suited to a ‘situated’ theory of learning, a trial and error method that is highly context dependent and hones flexibility and creativity. This form of knowledge is often regarded as intuition or context awareness.

Complexity assumes that a system is made up of individual agents, each acting to realize their own goals, and reacting to the broader system of consequences that result. However, in reality these agents are interdependent-following simple local rules, but affecting each other in a complex, coordinated fashion which continually changes the environment, which affects the system. Complexity reveals the difficulty inherent in prediction and control except in the short term. This challenges the concept that past behavior predicts future behavior. Accordingly, it is difficult to identify independent algorithms or ‘best practices’ which are not context-dependent or changeable over time.

Complex systems can exhibit unique properties that might simply be considered ‘perturbations’ from the perspective of other epistemologies. These include: *nonlinearity*-small changes in input can lead to large changes in output; *unintended consequences*-a result of emerging novelty and interdependence; *temporal displacement*-full effects may only be seen after several iterations as influences echo through the system; and *tipping points*-catastrophic state changes that are difficult to repair. For example, performance metrics are often examined in isolation (a reductionist perspective). If we attempt to optimize clinic productivity, while ignoring its complex interdependence with patient’s access to care and patient and provider satisfaction we may see a steady increase in production followed by a sudden turnover of providers or exodus of patients. Complex systems are influenced by the availability of resources in the environment, but cannot be determined by the environment.

With regard to educational tasks, complexity is best applied to situations where the learner requires adaptability in the face of uncertainty and change. A complexity epistemology should be reserved for higher level learners where the basics are familiar, they have experienced the limitations of decontextualized algorithms such as rules and guidelines, and both the clinical elements and conversation are novel or rapidly changing.

An example of the application of complexity philosophy would be a learning experience that emphasizes working within the care team to address a patient’s adherence difficulties. Adherence can be affected by many dynamic elements: economics, social support, employment, mood, motivation, family function, prior experience with the treatment, and cultural beliefs. If this team displayed effective support of patient adherence, the trainee working within this team might experience curiosity, attentiveness, and flexibility. They would learn each other’s strengths, weaknesses, and capabilities and function better as a team. Over time, a trainee may learn that despite effective teamwork, patient adherence is never assured.

## Learning Theories

We will now examine some contemporary learning theories from the perspective of these three epistemologies. These theories were selected as currently important representatives of three broad classes of learning theories: behavioral, cognitive, and social.

### Competency-based education (Behavioral)

Many health training systems have adopted competency-based education, essential elements of a competent practitioner, as a behavioral method for accountably documenting the quality of graduates. This frames learning goals as abstract competencies, more specific behavioral milestones, and entrustable professional activities (EPAs) that can help determine, for instance, what level of supervision is required for specific clinical activities. Competency-based education attempts to guide design and assessment using developmentally appropriate, specific, observable behaviors.

Most competencies are framed from a reductionist epistemology. For instance, the American Psychological Association competencies for primary care ( APA 2015) include “Utilizes knowledge about the effect of the family and other members of the support system on medical regimen adherence”. This may be useful because it is concrete and assumes that non-controversial stable entities (effects of support system) exist in the world independent of the observer. Although generally effective, reductionist competencies like this may have a subjective component that could be applied differently depending on irrelevant qualities of the trainee, such as impact on clinic staff ( Ginsberg et al 2010).

Some competencies are framed from a constructivist epistemology. This example comes from the Accreditation Council on Graduate Medical Education’s Next Accreditation System (ACGME 2015), “Manages patients with progressive responsibility and independence”. Here, trainee developmental expectations are globally stable but the trajectory for each individual trainee is unique and often dynamic, requiring comparison to a standardized trajectory through dialogue in a clinical competency committee.

Although rare, some competencies are framed from the complexity epistemology. For instance, the CanMEDS competencies (CanMEDS 2015) include “Recognize and respond to the complexity, uncertainty, and ambiguity inherent in medical practice”.

Selecting the correct epistemology from which to generate a competency is a matter of learner familiarity with the context (beginner favors reductionist, advanced favors complexity), stability of the situation (stability favors reductionist), and the degree of collective agreement about correct action (conflicting recommendations favor constructivist or complex philosophies).

### Clinical reasoning (Cognitive)

One common topic in health professional education is assessing and remediating deficiencies in clinical reasoning. Clinical reasoning involves complex interactions between knowledge, experience, performance, attention, emotion, and context. Several organizational structures for knowledge have been proposed such as semantic networks ( Bordage & Lemieux 1991), schemas ( van Kesteren et al 2014), and illness scripts (Norman & Rikers 2007). The accessibility of these organizational structures affects their usefulness for clinical reasoning. At a fundamental level, the formation and retrieval of these structures in memory are all impacted by dual processing theory ( Kahneman 2011). In this theory system one is automatic, rapid, and effortless (intuition). System two is analytic, slow, and requires attention (reason). Each system enables and constrains the other as they operate together.

Clinical reasoning from a reductionist epistemology is pedagogically useful for focusing on the elements required to develop a cognitive skill, such as interviewing patients. Skill development is a transition from advanced beginner, slow and fully attentive (such as following a check list-which embodies system two) to proficiency, rapidity and instinct (intuitive-which embodies system one). Reductionism provides guidance for this transition that includes a regular environment, opportunity for authentic practice, and rapid and unequivocal feedback. These strategies provide the emotional-neurochemical reinforcement (dopamine tuning) and augmentation of neuronal connections required for this instinctive learning to develop ( Roffman et al 2016).

From a constructivist epistemology, the content of cognitive states-thoughts, beliefs, memories and so on-are semantic. As such, they describe, through interconnected networks of words, the logically sufficient features of objects, such as ‘what is a chief complaint’. From these, we make causative inferences, such as ‘open-ended questions are initially more useful for eliciting chief complaints’. However, we do not do this in a vacuum. Concepts are affected internally by our neurophysiologic milieu (system 1) and externally as meanings are negotiated within communities (system 2) ( Rowlands 2010). Focusing from the constructivist perspective on clinical reasoning suggests the importance of training in authentic situations like simulations or Objective Structured Clinical Exams (OSCEs), which will involve realistic bodily responses, and jointly provide time to discuss and process performance.

Complexity epistemology adds a great deal to the understanding of clinical reasoning. Decision making in experts appears to be a “recognition primed” emergent property of nested systems ( Klein 1999). Experts must respond rapidly to important situations in dynamic contexts, which exhibit some regularity, but not complete predictability. These experts employ pattern matching intuition (system one) followed by deliberate mental simulation (system two) until the first potentially workable solution to the problem is identified and tested. These novel, adaptive solutions were not predictable and often are not explainable by the expert because they’re pre-conscious. The complexity perspective of clinical reasoning requires exposure to highly dynamic, real-life situations followed by exploration of tacit choices in order to make them more explicit.

From the clinical reasoning perspective, reductionist epistemology reveals useful concrete ‘rules’ that can be used to predictably enhance skill building. Constructivist epistemology can facilitate important discussion about theories of truth that require correspondence between our semantic descriptions (a collective network function) and features in the real world. Complexity epistemology reveals that some aspects of expert decision making (flexibility, creativity) may not be able to be taught, but rather must be experienced in dynamic, high-fidelity environments.

### Situated learning theory (Social)

Situated learning theory hypothesizes that individuals, activities, and the community are mutually constitutive-they determine themselves and each other over time. This theory, derived from direct observations of learners acquiring specific roles in a broad array of situations (midwives, tailors, quartermasters, butchers, and ‘dry’ alcoholics), reveals the extent to which ‘learning is doing’-or the importance of an apprentice-like approach to learning in communities of practice ( Lave & Wenger 1995).

From reductionist epistemology, situated learning theory can identify the specifics of the expected learner trajectory discussed above under competency-based education. These developmental milestones, consisting of concrete activities and community responsibilities, can anchor assessment and feedback. The individual can be serially compared to these norms to help set realistic expectations and to identify sudden deviations that might signal a new contextual issue that must be addressed, such as depression or substance abuse.

Constructivist epistemology explains the situated learning concept of “legitimate peripheral participation” (1995, pg. 29) -permission to provide a developmentally appropriate action in service to the overall group project (such as a student drawing blood during a ‘code blue’)-which allow the learner, through proximity, to observe expert practice and are seen to be critical to apprentice-like learning.

Complexity epistemology explains the unpredictable and never-ending process of identity formation within a community of practice through engagement, imagination, and alignment with community norms and goals.

Within situated learning theory, reductionist epistemology provides a useful template for expected development. Constructivist epistemology helps us to understand specifics about how the learning environment can help or hinder apprentice-like learning. Complexity theory highlights the evolutionary nature of professional identity formation.

## Application

To better understand how this could be useful for guiding interprofessional education, consider how it might apply to the following scenarios where you are asked to design training elements.

Scenario One: *You are associated with an interprofessional training clinic that has medical, nurse practitioner, and pharmacy residents and psychology post-doctoral fellows. As the psychology supervisor in the clinic, you are asked to design a motivational interviewing curriculum for all the clinic’s trainees to support behavior change in their patients.*

Early learners are often not ready for the full complexity of clinic and need simplified models that demand limited real-time processing and have concrete rules. This suggests that a reductionist epistemology would be most appropriate (e.g., motivational interviewing = open ended questions and ‘change talk’). Motivational interviewing (MI), in this context, is a skill you would like the learners to develop. Either a behavioral (performance checklist) or cognitive (skill development) learning theory alone or in combination would be appropriate. A behavioral performance checklist could be developed based on current ‘best practices’ for MI. Clinical reasoning (cognitive) suggests a regular training environment with opportunity for practice, such as a simulated patient, with rapid unequivocal feedback. Putting this all together, the psychology clinic director could pair with a primary care trainee (NP or medicine resident) to represent expertise in MI and a realistic view of the pressures of primary care practice. Together, they could develop a best-practice checklist and use this to prime and debrief a simulated patient encounter. Further, if this activity was coached by the psychology postdoctoral fellow, it could begin a trust relationship which could then evolve into coaching in real time with real patients, either by webcam or direct in-room observation.

Scenario Two: *You are asked to join a group that is establishing the structure and standards for an interprofessional team that will guide diagnosis and remediation of interprofessional ‘learners in difficulty’ from training programs in nurse practitioner, social work, medicine, pharmacy, and psychology.*

The requirement for structure suggests either a reductionist or constructivist epistemology would work best. You are being asked to evaluate and manage learners across professions within separate developmental frameworks. Both the interprofessional nature, suggesting a potential need to address power and conflict, and the expectation of an anticipated learning trajectory indicate that a social learning theory would work best. The critical element in this plan will be interprofessional faculty development. Creating a shared understanding of the expectations and typical trajectories for trainees in each discipline will be new to outside faculty members. Starting with a reductionist framework, comparing and contrasting existing profession-specific behavioral milestones would be optimal. This would allow faculty members across professions to gain an appreciation for each discipline’s unique learning trajectory and common points of difficulty. For instance, until we started working so closely together, the team physicians were unaware of a common developmental difficulty as nurse practitioner (NP) students transition from a ‘nurse’ role to a ‘provider’ role where a new level of uncertainty and responsibility is encountered in practice. Then, through interprofessional faculty dialogue (constructivist) these shared mental models could be refined into local standards based on the specific training opportunities available and learner expectations across training programs. One opportunity here is to design the process to include cross-professional mentoring, which will provide pragmatic knowledge of trajectories and common difficulties for the faculty and potentially be less threatening to the learners because their own training leadership are not directly involved. For instance, through this process a psychology faculty member might feel more comfortable aiding in the development of a remediation plan for a struggling pharmacy trainee, and might have uniquely helpful insights to offer. Reviewing taxonomy of learner difficulties (reductionist) could also inform the function of this team by suggesting general types of remediation plans that might be necessary to make available.

Scenario Three: *You are asked to develop a curriculum for advanced interprofessional learners to improve behaviors related to stereotypes and biases about each other.*

Requirements for this curriculum will parallel those espoused above in the introduction: finding a shared vocabulary, developing self-awareness, and learning to embrace differences. Finding a shared vocabulary can begin with an introductory exercise. The concreteness of a reductionist epistemology would be helpful at this stage. For instance, flip charts can be arranged around the room and profession-specific groups can be asked at each chart to share the ‘first phrase that comes to mind when I say the word ___________’ (own profession, such as ‘doctor’). Groups then move to the next flip chart and repeat the exercise with the next profession. They keep rotating until they return to their original chart and see what all the groups said about their profession (including themselves). A summary of these flip chart descriptions makes an excellent initial vocabulary for further discussion.

Greater self-awareness requires authentic situations with time for discussion and reflection so trainees can develop flexibility and adapt. Consciousness-raising activities should help the learner identify and accept tacit stereotypes and biases that could violate an espoused group norm and/or desired self-identity. For instance, implicit association tests ( Chung et al 2012) use descriptive lists and reaction times to infer bias between professions. These could bring to light and normalize differences in interprofessional assumptions. Discussions about these stereotypes will inevitably include the effects of power differentials and conflict resolution styles.

To allow trainees to internalize the value of differences, it is critically important that faculty role model participation in difficult conversations while maintaining rules of civil conversation. Faculty must also provide psychological safety by demonstrating empathy, warmth, respect and by suspending judgment (Gazda et al., 1982). These features suggest that a situated learning theory would be useful.

Adapting to this new awareness will require a series of ever-more-authentic situated learning exercises to eventually remodel professional identity. Starting from a reductionist perspective, one could begin by eliciting a vocabulary of stereotypical prototypes. Next, from a constructivist perspective, we could normalize stereotypes and biases using a set of video simulations portraying various stereotypes in use to generate discussion about the effects of those stereotypes on team function. Finally, applying a complexity perspective, we could use interprofessional role plays to help learners adapt when stereotypes are called to mind and to explore how to react and control them.

## Conclusions

Interprofessional training is multifaceted and is not adequately addressed by a one-size-fits-all set of learning assumptions and theories ( Braungart et al 2011). Early learners need relative concreteness, basic skill development, and feedback about their performance relative to expected norms. As trainees advance, the goals of the training program and the context take on more importance. These may range from the requirement for accountability and entrustment decisions based in direct observation of behavior, through the development of shared meaning and expectations by arranging for dialogue around divisive issues such as power differentials, to creating opportunities to reflect-in-action as the learner applies flexibility and creativity in novel, dynamic situations.

Although this approach may not make it explicit, selecting the correct tactic is not a one-person task. Negotiating this quagmire is difficult. Each of our professions has something to offer in addressing learning and designing and evaluating interprofessional curriculum. Interprofessional faculty cohesion is a foundational requirement for this process to work well. There must be faculty understanding of the principles described here and ‘permission’ given for interprofessional faculty to provide cross profession feedback, using the behavioral norms of the clinic as a guide.

We have found that working together and having a standardized approach to design of each curriculum element; based on trainee developmental level, program goals, and contextual needs, is very useful. Other epistemologies or learning theories could be added or substituted as needed in this model.

## Take Home Messages

• Epistemologies are assumptions about how the world is, how knowledge of the world can exist, and what procedures can distinguish between justified beliefs and opinions. Reductionism, Constructivism, and Complexity are synergistic epistemologies commonly used in health professional education.
• Learning theories hypothesize how knowledge is acquired, retained, and used. They guide instruction and assessment. They are often classified as behavioral, cognitive, or social theories.
• Selecting an appropriate epistemology and learning theory based on educational goals, learner development, and context is important for successful interprofessional curriculum development.

## Notes On Contributors

C. Scott Smith, MD is the national physician consultant for the Centers of Excellence in Primary Care Education and is a Professor of General Internal Medicine and Biomedical Informatics and Medical Education (Evaluation Division) at the University of Washington School of Medicine, Seattle, WA.

India King, PsyD is Associate Director for Evaluation, Boise Center of Excellence, VA Medical Center, Boise, ID and Affiliate Faculty, Idaho State University College of Pharmacy.

Amber Fisher, Pharm D is Co-Director of the Boise Center of Excellence, VA Medical Center, Boise, ID and Affiliate Faculty, Idaho State University College of Pharmacy.

William G. Weppner, MD, MPH is Co-Director of the Boise Center of Excellence, VA Medical Center, Boise, ID and Assistant Professor of General Internal Medicine, University of Washington, Seattle, WA.

Winslow G. Gerrish, PhD is Director of Behavioral Sciences, Research and Grants at the Family Medicine Residency of Idaho, Boise, ID and Assistant Professor of Psychology, University of Washington School of Medicine, Seattle, WA.
