# Supplementary material for: The Foundations of interprofessional curriculum. Finding the right epistemology and learning theory for the task
Source: MedEdPublish (2016). 2017 Jul 12;6:127. [Version 1] doi: 10.15694/mep.2017.000127 (PMC10885278; doi:10.15694/mep.2017.000127)
Supplement: Peer review history

# Reviewer report: Reviewer response for version 1

**DOI:** 10.21956/mep.19317.r28191

**Published:** 2017-07-28

**Authors:** Dujeepa D. Samarasekera

**Affiliations:** 1 National University of Singapore

This review has been migrated. The reviewer awarded 4 stars out of 5 Enjoyed reading the paper. The authors have nicely shared how best to base ones educational practices and course design on pedagogy. I feel that the paper adds value to education literature and gives a good insight to course developers on how best to construct educational activities.

Reviewer Expertise:

NA

No decision status is available

# Reviewer report: Reviewer response for version 1

**DOI:** 10.21956/mep.19317.r28189

**Published:** 2017-07-16

**Authors:** Neslihan Onder-Ozdemir

**Affiliations:** 1 The University of Sheffield

This review has been migrated. The reviewer awarded 3 stars out of 5 To design an ‘interprofessional curriculum’, three scenarios associated with training elements are reported very well in light of epistemologies, learning theories and over seven years of team-based clinical training experience. In line with the research setting and aim, situated learning is described with the key highlights: “apprenticeship” and learning as an aspect of “communities of practice” with the term “legitimate peripheral participation”. Like limitations with constructivism and problems with reductionism- as detailed in the manuscript, there are also some concerns with situated learning: insufficient attention to the community (in the present study ‘clinics’) where learners (‘trainees’) practise, which should be described. Indeed, in the present study, a deduction from three scenarios could be made (i.e., providing some details about the community where the research took place) to address the concern. For example, in the following take home message, the significance of context is reported. Thus, some references to the importance of community throughout the scenarios could enrich the content: “[…] Selecting an appropriate epistemology and learning theory based on educational goals, learner development, and context is important for successful interprofessional curriculum development". Also, proving information about potential problems that health care professionals may encounter while designing training elements could be useful.

Reviewer Expertise:

NA

No decision status is available

# Reviewer report: Reviewer response for version 1

**DOI:** 10.21956/mep.19317.r28190

**Published:** 2017-07-12

**Authors:** Trevor Gibbs

**Affiliations:** 1 AMEE

This review has been migrated. The reviewer awarded 3 stars out of 5 An interesting paper to read that clearly explains how learning theories and epistemologies can be applied to hopefully improving IPL. The various elements were well outlined by the authors and some clear examples were created to demonstrate application. However, I wonder how useful such a paper would be to those not already deeply involved with such theories and not being given such clear guidelines to what specific elements of IPL are to be taught and learned, such as those outlined in the three scenarios. I would imagine that many tasked with producing an IPL activity would start at a much broader, general level. It would be interesting to see how such theories and epistemologies are applied at that level

Reviewer Expertise:

NA

No decision status is available
